# Self-Subunit Swapping Occurs in Another Gene Type of Cobalt Nitrile Hydratase

**DOI:** 10.1371/journal.pone.0050829

**Published:** 2012-11-30

**Authors:** Yi Liu, Wenjing Cui, Yuanyuan Xia, Youtian Cui, Michihiko Kobayashi, Zhemin Zhou

**Affiliations:** 1 Key Laboratory of Industrial Biotechnology, Ministry of Education, School of Biotechnology, Jiangnan University, Wuxi, China; 2 Institute of Applied Biochemistry, and Graduate School of Life and Environmental Sciences, The University of Tsukuba, 1-1-1 Tennodai, Tsukuba, Ibaraki, Japan; Indian Institute of Science, India

## Abstract

Self-subunit swapping is one of the post-translational maturation of the cobalt-containing nitrile hydratase (Co-NHase) family of enzymes. All of these NHases possess a gene organization of <β-subunit> <α-subunit> <activator protein>, which allows the activator protein to easily form a mediatory complex with the α-subunit of the NHase after translation. Here, we discovered that the incorporation of cobalt into another type of Co-NHase, with a gene organization of <α-subunit> <β-subunit> <activator protein>, was also dependent on self-subunit swapping. We successfully isolated a recombinant NHase activator protein (P14K) of *Pseudomonas putida* NRRL-18668 by adding a Strep-tag N-terminal to the P14K gene. P14K was found to form a complex [α(StrepP14K)_2_] with the α-subunit of the NHase. The incorporation of cobalt into the NHase of *P. putida* was confirmed to be dependent on the α-subunit substitution between the cobalt-containing α(StrepP14K)_2_ and the cobalt-free NHase. Cobalt was inserted into cobalt-free α(StrepP14K)_2_ but not into cobalt-free NHase, suggesting that P14K functions not only as a self-subunit swapping chaperone but also as a metallochaperone. In addition, NHase from *P. putida* was also expressed by a mutant gene that was designed with a <β-subunit> <α-subunit> <P14K> order. Our findings expand the general features of self-subunit swapping maturation.

## Introduction

Metals commonly occur in proteins, modulating, if not underpinning, protein function [1]. It has been reported that more than 30% of structurally characterized proteins possess at least one metal ion [2]. Nitrile hydratase (NHase; EC 4.2.1.84) [3], which is composed of α- and β-subunits, contains either non-heme iron [4], [5] or non-corrinoid cobalt [6], [7] in the active site. NHase catalyzes the hydration of a nitrile to the corresponding amide, followed by the consecutive reactions: amide → acid → acyl-CoA, which are catalyzed by amidase [8] and acyl-CoA synthetase [5], [9], [10], respectively. Metal ions in both Co-NHase and Fe-NHase are located in their α-subunits, which share a characteristic metal-binding motif [CXLC(SO_2_H)SC(SOH)] containing two modified cysteine residues: cysteine-sulfinic acid (αCys-SO_2_H) and cysteine-sulfenic acid (αCys-SOH) [5], [11]–[15]. The apoenzyme is likely to be unmodified, based on the results of previous studies on NHase [16] and a related enzyme, thiocyanate hydrolase (SCNase) [17]–[19]. Non-corrinoid cobalt has received increasing interest not only in bioinorganic chemistry but also in biotechnology, and its availability and remarkable chemical versatility make it an invaluable catalyst in the chemical industry [20]–[23].

**Figure 1:**
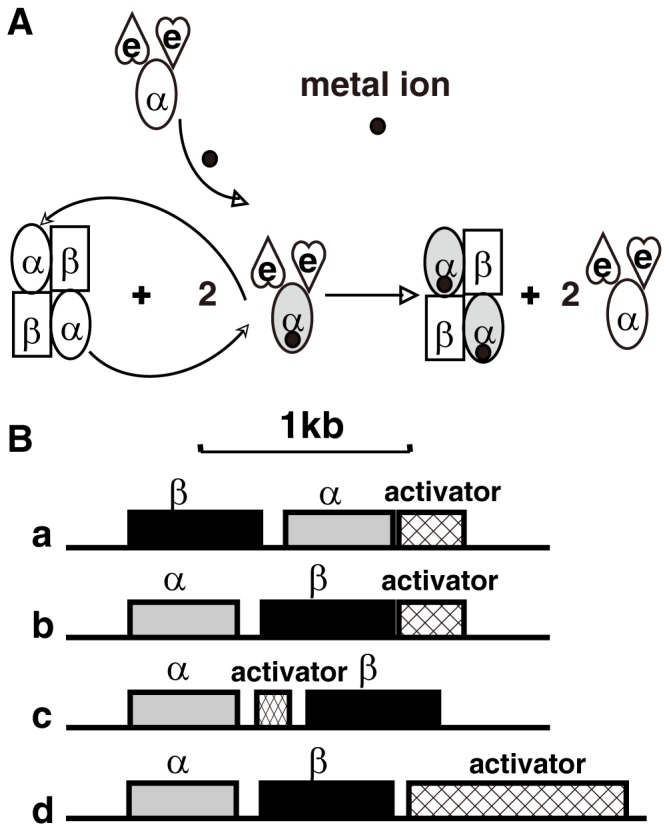
Self-subunit swapping and four types of NHases with different gene organizations. (A) Self-subunit swapping maturation of L-NHase. The cobalt ion is shown as a solid circle. The holo-α-subunit is shown in gray. (B) Four types of NHases with different gene organizations. a–c, three types of Co-NHases; d, Fe-NHase.

Metalloproteins have been characterized intensively for decades, yet only recently have investigators focused on the mechanisms of biological metallocenter assembly. The trafficking of metal ions into NHases is mediated by various “activator proteins” [1]. Fe-NHases require activators for functional expression in *Rhodococcus* sp. N-771 [24], *Pseudomonas chlororaphis* B23 [25], and *Rhodococcus* sp. N-774 [26]. A proposed metal-binding motif, CXCC, in the NHase activator of *Rhodococcus* sp. N-771 has been identified, and the activators for Fe-type NHases have been shown to act as metallochaperones [27]. Recently, a cobalt-containing NHase in *Rhodococcus rhodochrous* J1, which uses a novel mode of post-translational maturation, was discovered from studies on the low-molecular-mass NHase (L-NHase, encoded by the genes *nhlBA* and consisting of α_2_β_2_ subunits). This novel post-translational maturation involves the swapping of the metal-free α-subunit in the L-NHase with the cobalt-containing α-subunit of the L-NHase mediator, NhlAE (encoded by the genes *nhlAE* and consisting of αe_2_ subunits) (Fig. 1A) [28]. This post-translational maturation process is different from general mechanisms of metallocenter biosynthesis known thus far, and we have thus named it self-subunit swapping [28]. Compared with activators acting as metallochaperones in Fe-NHases, NhlE acts as a self-subunit swapping chaperone, exhibiting novel behavior for a protein in a protein complex. Furthermore, oxidation of the metal ligand cysteine residues and insertion of cobalt into the α-subunit occur in an NhlE-dependent manner [29]. Self-subunit swapping represents a new concept in the history of protein complex research. Besides L-NHase, self-subunit swapping maturation of the high-molecular-mass NHase (H-NHase) in *R. rhodochrous* J1 has also been confirmed [30]. Moreover, various other Co-NHases and an NHase family enzyme, SCNase, most likely maturate through self-subunit swapping based on identical features (i.e., enzyme properties, gene organizations, *etc.*) shared among these cobalt-containing enzymes [30].

NHase in *Pseudomonas putida* NRRL-18668 and acetonitrile hydratase (ANHase, an NHase that catalyzes the hydration of small aliphatic nitriles) from *Rhodococcus jostii* RHA1 are also Co-NHases in which P14K and AnhE are essential for their NHase maturation, respectively [31], [32]. However, their gene organizations are quite different from those of L-NHase and H-NHase. As shown in Fig. 1B, the structural genes of L-NHase and H-NHase have the order <β-subunit> <α-subunit> <self-subunit swapping chaperone>, while those in ANHase and the NHase of *P. putida* NRRL-18668 have the order <α-subunit> <AnhE> <β-subunit> [31] and <α-subunit> <β-subunit> <P14K> [32], respectively, the latter of which is identical to those of Fe-NHase except that the molecular weight of the metallochaperone in Fe-NHase is larger than that of P14K. While AnhE has been found to act as a metallochaperone (not as a self-subunit swapping chaperone) for cobalt incorporation into ANHase [31], the mechanism for cobalt incorporation into the NHase of *P. putida* NRRL-18668 remains unclear.

In the present study, we successfully isolated recombinant P14K and discovered that self-subunit swapping also occurs in the NHase of *P. putida* NRRL-18668, though its gene organization is quite different from those of L-NHase and H-NHase. Our findings expand the general features of self-subunit swapping maturation.

## Materials and Methods

### Bacterial Strain and Vector Plasmid

*E. coli* BL21 (DE3) was used as the host for the vector plasmid pET-24a(+), which was used for *A-B-P14K*, *A-B-’P14K*, *A-B-StrepP14K*, *HisT7A-B-P14K* and *B-A-P14K* expression.

### Construction of Plasmids

**Figure 2:**
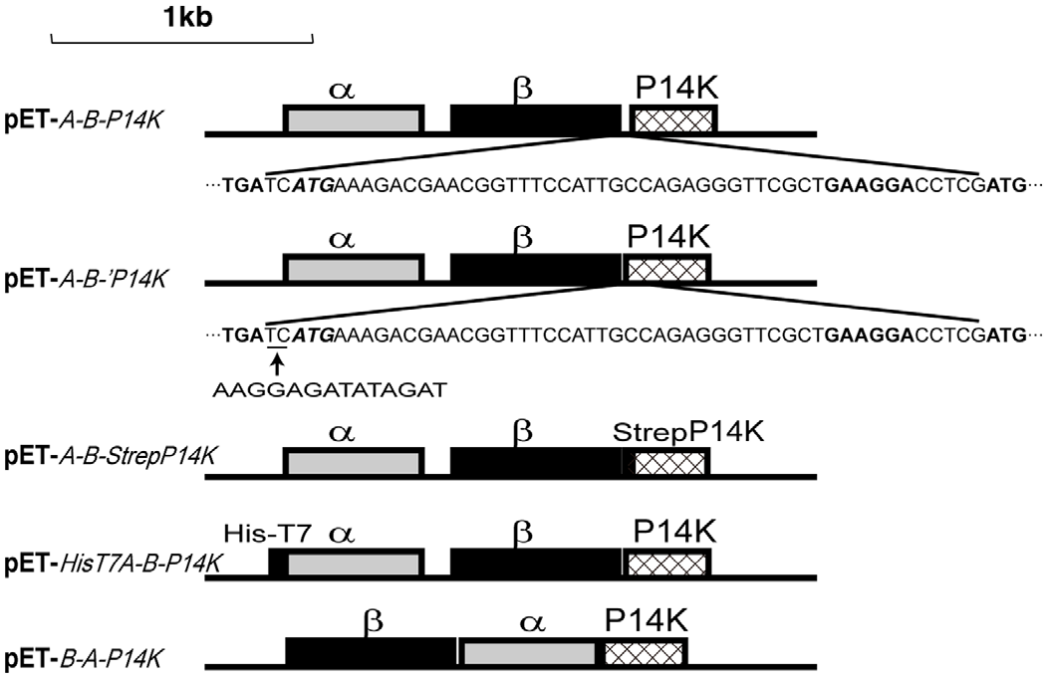
Plasmids used in this study. pET-*A-B-P14K*, a plasmid containing the α-subunit, β-subunit, and P14K genes; pET-*A-B-’P14K*, a plasmid containing the α-subunit, β-subunit, and the P14K genes with an enhanced RBS (the underlined nucleotide sequence was replaced with a strong RBS: AAGGAG) in which the italicized ATG was regarded as the start codon; pET-*A-B-StrepP14K*, a plasmid containing the α-subunit, β-subunit, and a Strep-Tagged P14K gene with an enhanced RBS; pET-*HisT7A-B-P14K*, a plasmid containing a His-T7-Tagged α-subunit gene, the β-subunit, and P14K genes; pET-*B-A-P14K*, a plasmid containing the NHase and P14K genes with a <β-subunit> <α-subunit> <P14K> order.

Genomic DNA from *P. putida* NRRL-18668 was isolated and used to clone the NHase and P14K genes, denoted as *A-B-P14K*, with the primers A-up and P14K-down (Table 1). The PCR products were digested with NdeI and EcoRI, ligated into pET-24a(+) to construct pET-*A-B-P14K* (Fig. 2), transformed into *E. coli* JM109, and sequenced. The clones with the correct sequences were transformed into *E. coli* BL21 (DE3). An overlap extension PCR protocol was used to construct the plasmid pET-*A-B-’P14K*. Two PCRs, with the primer pairs A-up and B-down(rbs), P14K-up(rbs) and P14K-down (Table 1), and the plasmid pET-*A-B-P14K* as the template, were performed for the first round. These reactions produced 5' and 3' fragments of *A-B-’P14K*, respectively. The second round of PCR was performed by mixing equimolar amounts of the first-round products, followed by amplification using the primers A-up and P14K-down to produce the full-length *A-B-’P14K*. The subsequent procedures were identical to those used to construct pET-*A-B-P14K*. Plasmid pET-*A-B-StrepP14K* was constructed in the same manner as pET-*A-B-’P14K,* using the primer pairs A-up with B-down(Strep) (Table 1) and P14K-up(Strep) (Table 1) with P14K-down. Plasmid pET-*HisT7A-B-P14K* was constructed in the same manner as pET-*A-B-P14K,* using the primer pairs HisT7A-up (Table 1) and P14K-down. Plasmid pET-*B-A-P14K* was constructed in the same manner as pET-*A-B-’P14K* except that three fragments of *A-B-’P14K* were produced in the first-round, using the primer pairs B-up and B-down(BA), A-up(BA) and A-down(AP), and P14K-up(AP) and P14K-down (Table 1).

**Table 1:** Oligonucleotide primers used in this study.

Name	Sequence
A-up	5′-GGAATTC*CATATG*GGGCAATCACACACGC-3′
P14K-down	5′-CCG*GAATTC*TCAAGCCATTGCGGCAACGA-3′
B-down(rbs)	5′-**CATATCTATATCTCCTTTCA**CGCTGGCTCCAGGTAGTC-3′
P14K-up(rbs)	5′-**TGAAAGGAGATATAGATATG**AAAGACGAACGGTTTC-3′
B-down(Strep)	5′-**GCGGGTGGCTCCAGCTT**GCCATATCTATATCTCCTTTCACGCTGGCTCCAGGTAGTCATC-3′
P14K-up(Strep)	5′-**AAGCTGGAGCCACCCGC**AGTTCGAAAAGGGTGCAAAAGACGAACGGTTTCCATT-3′
HisT7A-up	5′-GGAATTC*CATATG*GGCAGCAGCCATCATCATCATCATCACAGCAGCGGCCTGGTGCCGCGCGGCAGCCATATGGCTAGCATGACTGGTGGACAGCAAATGGGTCGCGGGCAATCACACACGCATGACCAC-3′
B-up	5′-GGAATTC*CATATG*AATGGCATTCACGATACT-3′
B-down(BA)	5′-**CATATCTATATCTCCTTTCA**CGCTGGCTCCAGGTAGTC-3′
A-up(BA)	5′-**TGAAAGGAGATATAGATATG**GGGCAATCACACACGC-3′
A-down(AP)	5′-**CATATCTATATCTCCTTTTA**ATGAGATGGGGTGGGTT-3′
P14K-up(AP)	5′-**TAAAAGGAGATATAGATATG**AAAGACGAACGGTTTC-3′

Italicized letters denote the NdeI (in A-up, HisT7A-up, and B-up) and EcoRI (in P14K-down) restriction sites, respectively; bold letters indicate the overlapping nucleotides; underlined letters indicate the His-Tag and T7-Tag, respectively.

### Culture Conditions

*E. coli* BL21 (DE3) transformants containing the respective plasmid constructs were grown at 37°C in TB medium (1.2% (w/v) tryptone, 2.4% (w/v) yeast extract, 0.4% (v/v) glycerol, 17 mM KH_2_PO_4_, and 72 mM K_2_HPO_4_) containing CoCl_2_^.^6H_2_O (0.05 g/liter) and kanamycin (50 µg/ml) until the *A*_600_ reached 0.8. Isopropyl β-D-thiogalactopyranoside was added to a final concentration of 0.4 mM, and the cells were incubated at 24°C for 16 h.

### Purification of Enzymes

All purification steps were performed at 0–4°C. All procedures, except ammonium sulfate precipitation, were conducted with an AKTA purifier (GE Healthcare UK Ltd.). Potassium phosphate buffer (KPB) (10 mM, pH 7.5), containing 0.5 mM dithiothreitol (DTT), was used in all purification steps.

Cell extract preparation was performed as previously described. Centrifugation was conducted for 20 min at 18000×*g*. NHase was partially purified by ammonium sulfate fractionation (35–65%) followed by dialysis against KPB. The dialyzed solution was applied to a DEAE-Sephacel column (3×5 mL) (GE Healthcare UK Ltd.) equilibrated with KPB. We eluted the protein from the column with a 1 L linear gradient from 0 to 0.5 M KCl in KPB. The resulting partially purified enzyme was pooled, and ammonium sulfate was then added to obtain 70% saturation. After centrifugation of the suspension, the precipitate was dissolved in KPB containing 0.2 M KCl followed by application to a HiLoad 16/60 Superdex 200 prep grade column (GE Healthcare UK Ltd.) that was equilibrated with the 0.2 M KCl-containing KPB. The enzyme that was eluted from the HiLoad 16/60 Superdex 200 prep grade column was precipitated with ammonium sulfate (70% saturation) followed by dialysis against KPB. The dialyzed solution was applied to a Resource Q column (6 mL) (GE Healthcare UK Ltd.) equilibrated with KPB. We eluted proteins from the column with a 1 L linear gradient from 0 to 0.5 M KCl in KPB. α(StrepP14K)_2_ was purified with a StrepTrap HP column (GE Healthcare UK Ltd.) equilibrated with KPB. We eluted proteins from the column with KPB containing 25 mM desthiobiotin. The resulting partially purified enzyme was pooled and further purified with a HiLoad 16/60 Superdex 200 prep grade column (GE Healthcare UK Ltd.) in the same manner as NHase. The fractions containing the enzymes during the purification steps were revealed by SDS-PAGE, and a sequence analysis of N-terminal amino acids was performed at the final step.

### Enzyme Assay

NHase activity was assayed in a reaction mixture (0.5 ml) containing 10 mM KPB (pH 7.5), 20 mM 3-cyanopyridine, and 10 µl of enzyme containing activation buffer or an appropriate amount of the enzyme. The reaction was performed at 20°C for 20 min and stopped with the addition of 0.5 ml of acetonitrile. The amount of nicotinamide formed in the reaction mixture was determined as previously described. One unit of NHase activity was defined as the amount of enzyme that catalyzed the release of 1 µmol of nicotinamide per min at 20°C.

### Determination of Cobalt Ion Incorporation in the Purified Enzymes

Enzymes were dialyzed against 1 mM KPB (pH 7.5). After dialysis, the enzymes, at a concentration of 0.5 mg/ml, were analyzed with a Shimadzu AA-7000 atomic absorption spectrophotometer under the following conditions: wavelength, 240.73 nm; lamp current, 12 mA; and slit width, 0.2 nm.

### Molecular Mass Determination of the Purified Proteins

A HiLoad 16/60 Superdex 200 prep grade column (GE Healthcare UK Ltd.) equilibrated with 10 mM KPB (pH 7.5) containing 0.2 M KCl was used to estimate the molecular masses of the purified NHase and α(StrepP14K)_2_. This step was conducted with an AKTA purifier (GE Healthcare UK Ltd.) at 0–4°C, with a flow rate of 0.5 mL/min.

### Conversion of apo-NHase to holo-NHase by holo-α(StrepP14K)_2_

The purified cobalt-free NHase (apo-NHase) (from a culture in the absence of cobalt ion) (final concentration, 0.1 mg/ml) was mixed with the purified cobalt-containing α(StrepP14K)_2_ [holo-α(StrepP14K)_2_] (final concentration, 0.8 mg/ml) followed by incubation in 10 mM KPB (pH 7.5) at 24°C. As a control, the purified apo-NHase was mixed with cobalt ion (20 µM) without holo-α(StrepP14K)_2_ and incubated in the same manner.

### Incorporation of Cobalt into the α(StrepP14K)_2_

The cobalt-free α(StrepP14K)_2_ [apo-α(StrepP14K)_2_] from transformed cells containing pET-*A-B-StrepP14K* cultured in the absence of cobalt was purified. The apo-α(StrepP14K)_2_ (0.1 mg/ml, final concentration) was mixed with cobalt (final concentration, 20 µM) and GSH (1 mM) in 10 mM KPB (pH 7.5) followed by incubation at 24°C for 4 h, and then purified the reconstituted α(StrepP14K)_2_ as [R-α(StrepP14K)_2_].

### Analytical Methods

UV-Vis spectra were obtained with a U-0080D spectrophotometer (Hitachi, Tokyo, Japan) at room temperature. Enzymes were dialyzed against 10 mM KPB (pH 7.5), and 1.0 mg/ml samples were prepared. N-terminal sequencing was performed on samples electroblotted onto a polyvinylidene difluoride membrane after SDS-PAGE using an ABI PROCISETM 492cLC sequencer by Shanghai Gene Core Biotechnologies Co., Ltd.

## Results and Discussion

### Identification of the P14K Gene of P. Putida NRRL-18668

**Figure 3:**
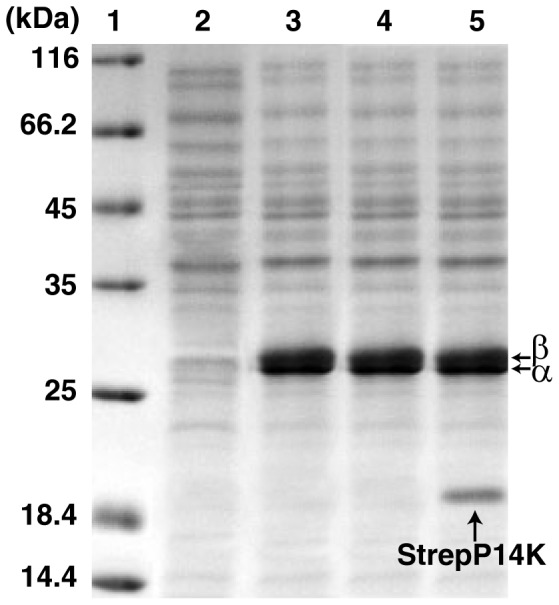
SDS-PAGE and NHase activity of cell-free extract of each *E. coli* BL21 (DE3) transformant. 1, markers; 2, control; 3, transformant containing pET-*A-B-P14K*; 4, transformant containing pET-*A-B-’P14K*; 5, transformant containing pET-*A-B-StrepP14K*.

To clarify the mechanism of cobalt incorporation into the NHase of *P. putida* NRRL-18668, we attempted to isolate P14K, which is essential for NHase activity [32]. P14K was hardly detected on SDS-PAGE (Fig. 3), which is consistent with a previous report [32]. To enhance the expression of P14K, the potential ribosome-binding site (RBS) (GAAGGA) [32] was substituted by an enhanced RBS (AAGGAG). However though two bands corresponding to α- and β-subunits of the NHase were observed on SDS-PAGE, NHase activity in the cell-free extracts was very low.

**Figure 4:**
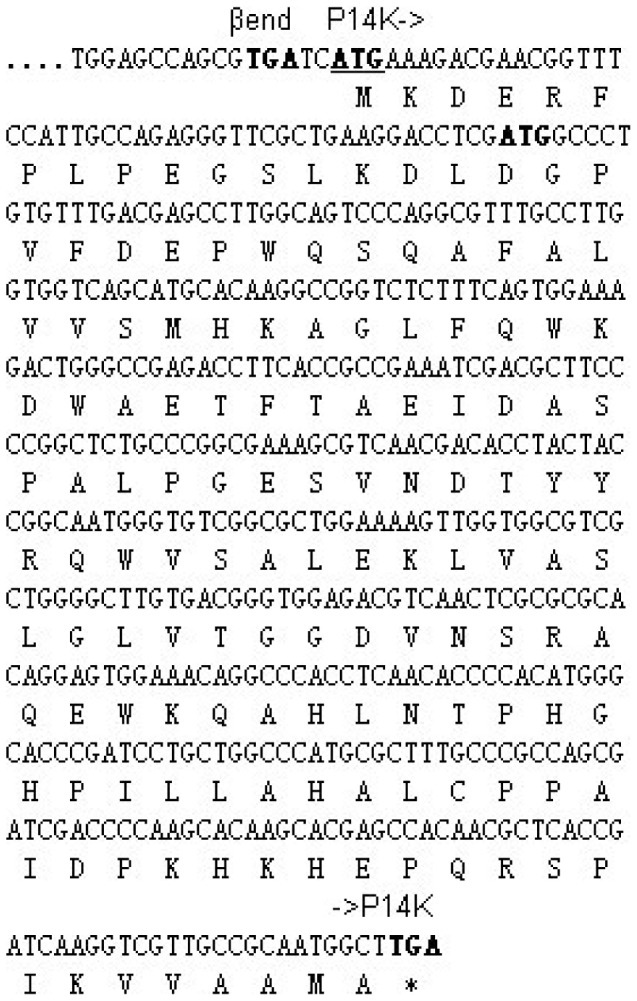
Sequence of P14K from *P. putida* NRRL-18668. The nucleotide sequence of the region downstream of the NHase *B* gene and the deduced amino acid sequence. The first underlined ATG in the nucleotide sequence is confirmed to be a genuine start code.

We found a second ATG that is located 49 bp upstream of the putative start codon (ATG) of the P14K gene (Fig. 4). We assumed that this preceding ATG is the actual start codon of P14K, and therefore a plasmid (pET-*A-B-’P14K*) (Fig. 2) including the NHase genes, the enhanced RBS, and the P14K gene beginning from this first ATG was constructed and used for NHase and P14K expression. Although P14K was not detected on SDS-PAGE, the NHase activity in the cell-free extracts from cells expressing pET-*A-B-’P14K* was similar to that from cells expressing pET-*A-B-P14K* (120.5 U/mg). P14K was successfully expressed when a Strep-tag was added to the N-terminus of the P14K gene in pET-*A-B-P14K* (resulting in pET-*A-B-StrepP14K*) (Fig. 2), and the NHase activity (118.3 U/mg) in the cell-free extracts from cells expressing pET-*A-B-StrepP14K* was at the same level as that from cells expressing pET-*A-B-P14K*. These findings suggested that the actual start codon of the P14K gene, encoding a 16.0 kDa protein, is only 2 bp downstream of the stop codon of the β-subunit gene and that the amino acid sequence for P14K is longer by 16 amino acids than has been previously reported (Fig. 4).

### Isolation of a Cobalt-containing Mediator, α(StrepP14K)_2_

**Figure 5:**
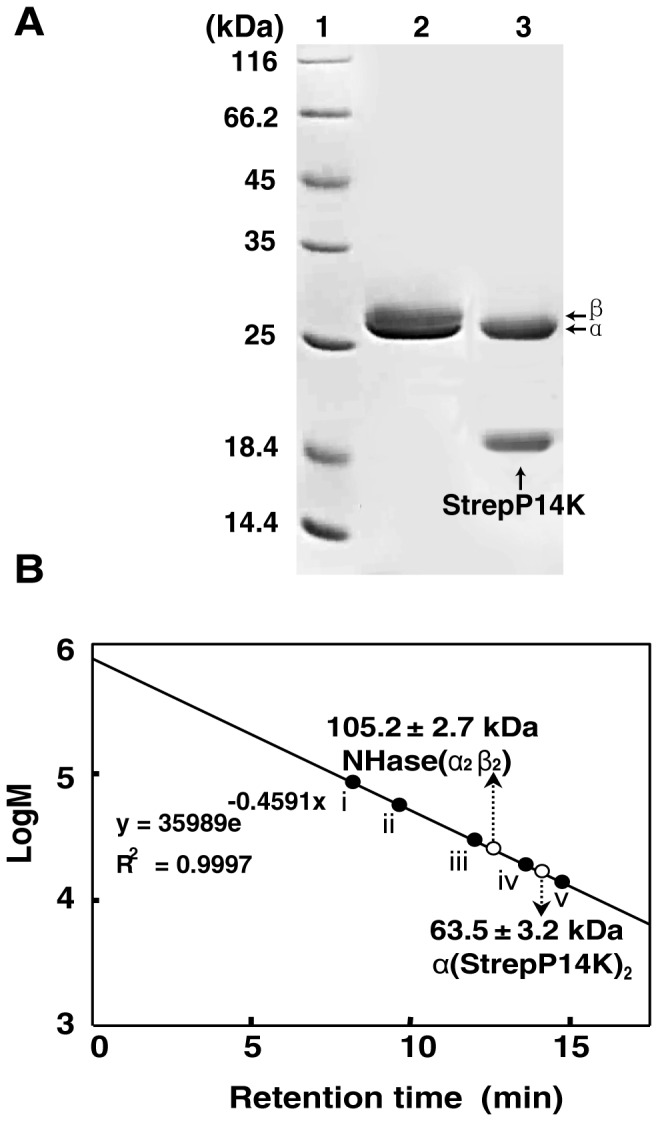
Purification of NHase and P14K. (A) SDS-PAGE of purified NHase and P14K. (B) Determination of the molecular mass and structures of the NHase and the complex of the α-subunit and P14K on a Superdex 200 prep grade column. Standard proteins used for the molecular mass determination are as follows: (i) thyroglobulin (bovine thyroid) (669 kDa); (ii) ferritin (horse spleen) (440 kDa); (iii) aldolase (rabbit muscle) (158 kDa); (iv) conalbumin (chicken egg white) (75 kDa); and (v) ovalbumin (hen egg) (43 kDa). The molecular mass determined is shown by an open circle. The values obtained from gel filtration analysis represent the means±SD for at least three independent experiments.

**Figure 6:**
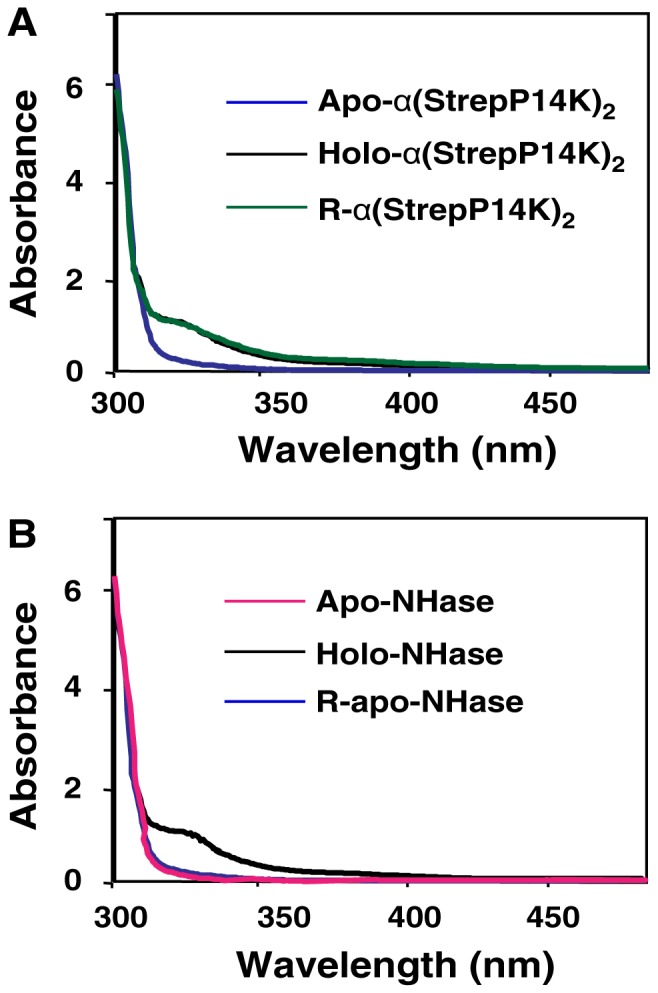
UV-Vis absorption spectra of the purified α(StrepP14K)_2_ (A) and NHases (B). (A) UV-Vis absorption of α(P14K)_2_ [apo-α(StrepP14K)_2_, holo-α(StrepP14K)_2_, and R-α(StrepP14K)_2_]. (B) UV-Vis absorption of NHases (apo-α_2_β_2_, holo-α_2_β_2_, and R-apo-α_2_β_2_).

To clarify the role of P14K in the activation of NHase, we purified the gene product (P14K) from the transformant harboring pET-*A-B-StrepP14K*. As a result, P14K was confirmed to form a complex with the α-subunit of NHase (Fig. 5A) by gel filtration analysis and protein N-terminal sequence analysis (α-subunit, GQSHTHD; StrepP14K, ASWSHPQ). The molecular mass of the complex of the α-subunit and P14K was determined to be 63.5 kDa by gel filtration analysis (Fig. 5B). Because the calculated molecular masses of the α-subunit and StrepP14K are 23.2 kDa and 17.3 kDa, respectively, the subunit composition of the complex should be α(StrepP14K)_2_. The purified α(StrepP14K)_2_ was found to contain cobalt [0.91 mol/mol of α(StrepP14K)_2_] and exhibited no NHase activity (Table 2). The UV-Vis spectrum of α(StrepP14K)_2_ showed an extra shoulder in the 300–350 nm region (Fig. 6A), which is similar to the cobalt-containing NHase (holo-NHase) (Fig. 6B), suggesting that the Co-ligand environment of α(StrepP14K)_2_ is identical to the holo-NHase.

**Table 2:** Characterization of the purified NHases, α(StrepP14K)_2_, R-NHases and R-α(StrepP14K)_2_.

Protein (plasmid)	NHase activity, units/mg	Co content (mol of ions/mol of protein)	N-terminal sequence of α-subunit
Holo-NHase (pET-*A-B-P14K*)	439±3.6	0.88±0.06/αβ	GQSHTHD
Apo-NHase (pET-*A-B-P14K*)	18.3±3.2	0.03±0.01/αβ	ND*
Holo-NHase (pET-*A-B-StrepP14K*)	421±6.0	0.89±0.05/αβ	ND
Holo-α(StrepP14K)_2_ (pET-*A-B-StrepP14K*)	0	0.91±0.01/α(StrepP14K)_2_	GQSHTHD
Apo-α(StrepP14K)_2_ (pET-*A-B-StrepP14K*)	0	0.04±0.01/α(StrepP14K)_2_	ND
R-apo-NHase	418±5.8	0.87±0.05/αβ	ND
R-α(StrepP14K)_2_ (pET-*A-B-StrepP14K*)	0	0.90±0.04/α(StrepP14K)_2_	ND
Holo-NHase_(HisT7-α)_ (pET-*HisT7A-B-P14K*)	396±8.7	0.89±0.05/αβ	ND
Apo-NHase_(HisT7-α)_ (pET-*HisT7A-B-P14K*)	20.5±4.9	0.04±0.02/αβ	GSSHHHH
R-NHase_(HisT7-α)_	417±8.0	0.87±0.03/αβ	GQSHTHD
Holo-NHase (pET-*B-A-P14K*)	410±6.3	0.89±0.05/αβ	ND

* ND, not detected.

The corresponding expression plasmids are shown in parentheses.

### Conversion of apo-NHase to holo-NHase by holo-α(StrepP14K)_2_

**Figure 7:**
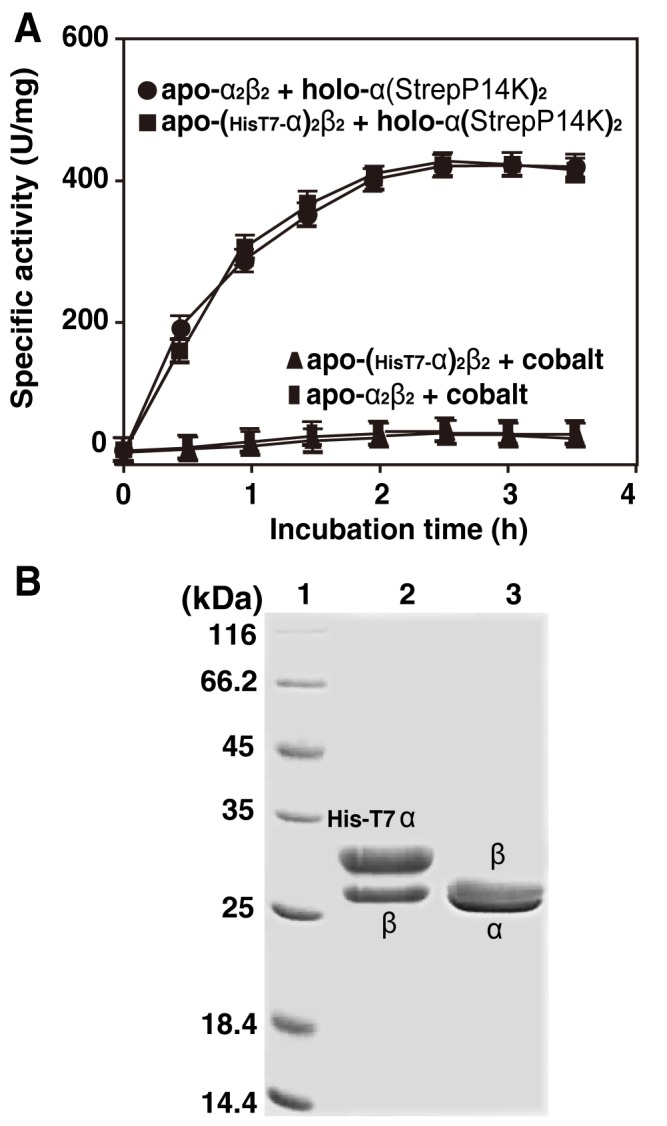
Post-translational activation of apo-NHase by holo-α(StrepP14K)_2_. (A) Activation of apo-NHase and apo-NHase_(HisT7-α)_ by holo-α(StrepP14K)_2_. Apo-NHase or apo-NHase_(HisT7-α)_ (final concentration, 0.1 mg/ml) was incubated with the purified holo-α(StrepP14K)_2_ (final concentration, 0.8 mg/ml) in 10 mM KPB (pH 7.5) at 24°C. (B) SDS-PAGE of apo-NHase_(HisT7-α)_ and R-apo-NHase_(HisT7-α)_. 1, markers; 2, apo-NHase_(HisT7-α)_; 3, purified R-apo-NHase_(HisT7-α)_.

The P14K is necessary for functional NHase expression and forms a complex with the α-subunit as α(StrepP14K)_2_, which is very similar to NhlE and NhhG in *R. rhodochrous* J1, both of them form a complex with the responsible α-subunit of L- and H-NHases as NhlAE (αe_2_) and NhhAG (αg_2_) [28], [30]. NhlAE and NhhAG have been reported to incorporate cobalt ions into cobalt-free L-NHase and H-NHase, respectively, during their post-translational maturation, resulting in the formation of cobalt-containing L-NHase and H-NHase [28], [30]. To elucidate the role of the α(StrepP14K)_2_ in the formation of an active NHase from *P. putida* NRRL-18668, the purified apo-NHase was mixed with the purified holo-α(StrepP14K)_2_ followed by incubation at 24°C as described in **EXPERIMENTAL PROCEDURES**. NHase activity in the mixture was found to increase as the incubation time increased, while no significant activity increasing was observed in the control (Fig. 7A). We then purified the reconstituted NHase (R-apo-NHase) from the mixture and found that its activity (418 U/mg) and cobalt content (0.87 mol/mol of αβ) (Table 2) were similar to those of the cobalt-containing NHase (holo-NHase) (Table 2). Like holo-NHase, R-NHase also exhibited an extra shoulder in the 300–350 nm region (Fig. 6B). These results suggest that the Co-ligand environment of R-NHase is identical to that of holo-NHase. Together with the incorporation of cobalt in R-NHase, these results thus demonstrate that α(StrepP14K)_2_ participates in the post-translational maturation of NHase.

### Self-subunit Swapping in the NHase of *P. putida* NRRL-18668

To determine the source of the α-subunit in R-NHase, we designed a plasmid containing a HisT7-Tag, *HisT7A-B-P14K*, in which a HisT7-Tag was added N-terminal to the α-subunit of NHase to distinguish between the apo-NHase and α(StrepP14K)_2_ α-subunits. The plasmid pET-*HisT7A-B-P14K* (Fig. 2) was constructed and expressed, and the HisT7-Tagged apo-NHase [apo-NHase_(HisT7-α)_] was purified. We mixed apo-NHase_(HisT7-α)_ with holo-α(StrepP14K)_2_, confirmed the post-translational activation of NHase *in vitro*; as the control, no significant activity increasing was observed in mixture of apo-NHase_(HisT7-α)_ and cobalt ion (Fig. 7A). The reconstituted NHase [R-NHase_(HisT7-α)_] from the mixture of apo-NHase_(HisT7-α)_ with holo-α(StrepP14K)_2_ was purified. SDS-PAGE analysis indicated that the α-subunit in the R-NHase_(HisT7-α)_ was different from that in NHase_(HisT7-α)_ (Fig. 7B); furthermore, N-terminal amino acid sequence analysis indicated that the α-subunit of the R-NHase_(HisT7-α)_ was a HisT7-Tag free α-subunit (Table 2), demonstrating that the apo-α-subunit of apo-NHase_(HisT7-α)_ was replaced by the holo-α-subunit of holo-α(StrepP14K)_2_. These findings revealed that the incorporation of cobalt into apo-NHase from holo-α(StrepP14K)_2_ depended on self-subunit swapping, resulting in the formation of holo-NHase, and that P14K acted as a self-subunit swapping chaperone analogous to NhlE for L-NHase and NhhG for H-NHase.

Self-subunit swapping has been found to be a general maturation mechanism in various Co-NHases and an NHase family enzyme, SCNase [30]. All of these enzymes share the same features: (*i*) self-subunit swapping chaperone genes are located just downstream of the structural genes; (*ii*) the structural genes for the α- and β-subunits are in the order of β and α; (*iii*) the molecular weight of the self-subunit swapping chaperone is less than 17 kDa; (*iv*) the self-subunit swapping chaperones exhibit weak sequence similarity to the NHase β-subunits; and (*v*) all of these small proteins lack metal-binding motifs known thus far [30]. Although P14K shows significant sequence similarity to these self-subunit swapping chaperones (more than 30%) and the two share similar features, the structural gene order for the α- and β-subunits of NHase from *P. putida* NRRL-18668 is quite different from those of the various Co-NHases. However, in this work, we discovered that self-subunit swapping also occurred in the NHase of *P. putida* NRRL-18668, demonstrating that cobalt incorporation into other NHases with an <α-subunit> <β-subunit> gene order [such as NHase in *Bordetella petrii* DSM 12804 [33] and *Comamonas testosteroni* 5-MGAM-4D [34]] is also dependent on self-subunit swapping. These findings reveal that self-subunit swapping does not depend on the gene order of the α- and β-subunit, expanding the general features of self-subunit swapping maturation.

### P14K as a Metallochaperone

We previously reported that cobalt is directly inserted into the apo-α-subunit of apo-NhlAE and apo-NhhAG in the presence of a reducing agent [DTT, 2-mercaptoethanol or glutathione (GSH)] *in vitro*, suggesting that NhlE and NhhG are also metallochaperones that are crucial for cobalt insertion into the α-subunit [29]. To elucidate the role of P14K in the incorporation of cobalt into the α-subunit of NHase from *P. putida* NRRL-18668, the purified apo-α(StrepP14K)_2_ from transformed cells containing pET-*A-B-StrepP14K* cultured in the absence of cobalt was mixed with cobalt followed by incubation as described in **EXPERIMENTAL PROCEDURES**. The R-α(StrepP14K)_2_ was purified, and its cobalt content was compared with that of holo-α(StrepP14K)_2_ and apo-α(StrepP14K)_2_. The determination of cobalt content showed that R-α(StrepP14K)_2_ contained 0.90 mol ion/mol of α(StrepP14K)_2_ (Table 2), which is similar to the cobalt content (0.91 mol ion/mol of α(StrepP14K)_2_) of holo-α(StrepP14K)_2_ (Table 2). The UV-Vis spectrum of R-α(StrepP14K)_2_ was similar to that of holo-α(StrepP14K)_2_ but not to that of apo-α(StrepP14K)_2_. As shown in Fig. 6A, an extra shoulder in the 300–350 nm region was found for R-α(StrepP14K)_2_ and holo α(StrepP14K)_2_. R-α(StrepP14K)_2_ was also able to convert apo-NHase into holo-NHase (data not shown). These findings suggested that cobalt was directly incorporated into apo-α(StrepP14K)_2_, resulting in the formation of holo-α(StrepP14K)_2_. Additionally, as seen in apo-L-NHase and apo-H-NHase [28], [30], cobalt was not directly incorporated into apo-NHase (data not shown). These findings demonstrated that a cobalt ion was directly incorporated into the apo-α-subunit of apo-α(StrepP14K)_2_ but not into that of apo-NHase *in vitro* and that P14K is involved in cobalt incorporation as a metallochaperone, which is similar to that of NhlE and NhhG in *R. rhodochrous* J1.

### Influence of the α- and β-subunit Gene Order in the NHase Gene Cluster on NHase Biosynthesis

**Figure 8:**
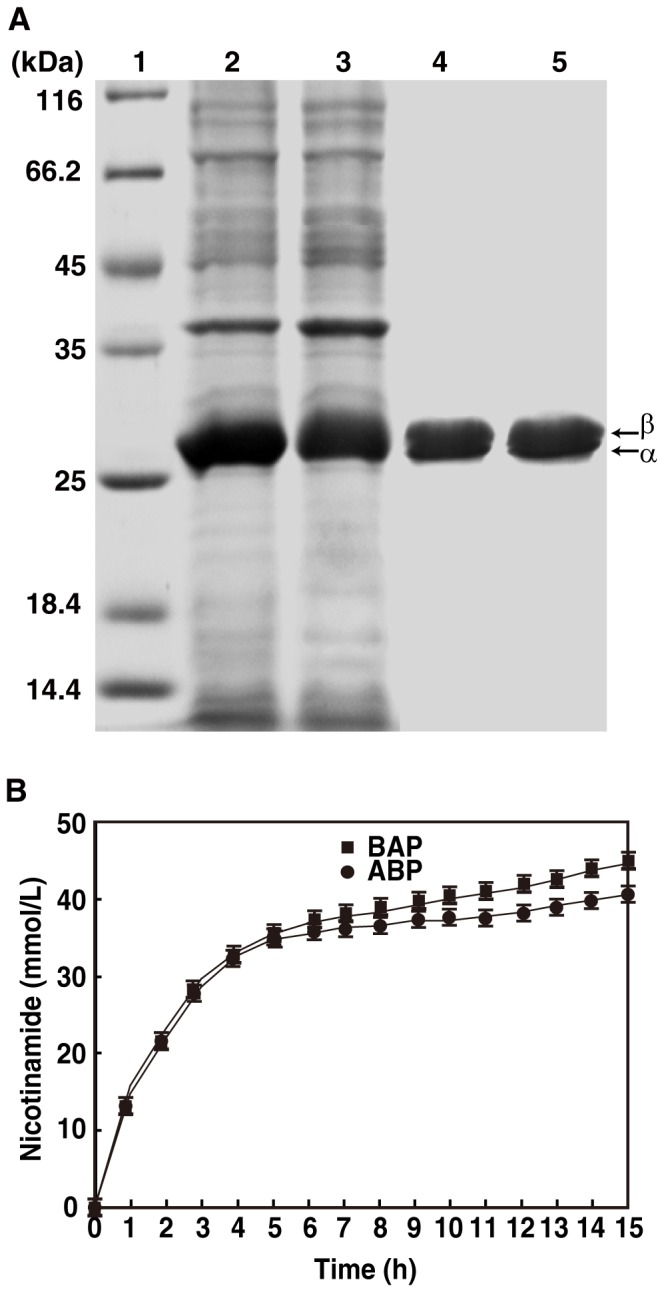
Comparison of NHases encoded by the *A-B-P14K* and *B-A-P14K* genes. (A) Expression and purification of NHases encoded by the *A-B-P14K* and *B-A-P14K* genes. 1, markers; 2, cell extract of the transformant containing pET-*A-B-P14K*; 3, cell extract of the transformant containing pET-*B-A-P14K*; 4, purified NHase from the transformant containing pET-*A-B-P14K*; 5, purified NHase from the transformant containing pET-*B-A-P14K*. (B) Catalytic efficiency of the two NHases encoded by *B-A-P14K* was a little superior to that encoded by *A-B-P14K.* The two transformants harboring pET-*A-B-P14K* and pET-*B-A-P14K* were incubated under the same condition as described in **EXPERIMENTAL PROCEDURES,** respectively, high concentration of 3-cyanopyridine (430 mM, final) was added after IPTG addition. The values represent the means±SD for at least triplicate independent experiments.

Although the α- and β-subunit gene organization of NHase in *P. putida* NRRL-18668 is quite different from those of L-NHase and H-NHase, the cobalt incorporation into the NHase of *P. putida* NRRL-18668 was found to be entirely dependent on self-subunit swapping. This finding raises the question as to whether NHase from *P. putida* NRRL-18668 could also be synthesized even when the gene organization is <β-subunit> <α-subunit> <P14K>. We designed a mutant gene, *B-A-P14K*, in which the gene organization for the α- and β-subunits of NHase from *P. putida* NRRL-18668 was changed to <β-subunit> <α-subunit> <P14K>, which is identical to that of L-NHase and H-NHase, and constructed pET-*B-A-P14K* (Fig. 2). The transformant harboring pET-*B-A-P14K* was used for NHase expression. NHase encoded by *B-A-P14K* was successfully expressed at the same level as that of *A-B-P14K* (Fig. 8A), and the specific activity and cobalt content of the NHase encoded by *B-A-P14K* was identical to that of *A-B-P14K* (Table 2). These findings demonstrate that the gene order for the α- and β-subunits does not influence the biosynthesis of NHase from *P. putida* NRRL-18668. Subsequently, catalytic efficiency of the two NHases encoded by *B-A-P14K* and *A-B-P14K* was compared. The two transformants harboring pET-*A-B-P14K* and pET-*B-A-P14K* were incubated under the same condition with high concentration of 3-cyanopyridine (430 mM), the production of the nicotinamide in the two cultures were measured as the incubation time. It was interesting that though large amount of nicotinamide was observed in the two cultures, the concentration of nicotinamide in the culture of transformants harboring pET-*B-A-P14K* was slightly higher than that of the transformant harboring pET-*A-B-P14K* at the same time (Fig. 8B), indicating that the catalytic efficiency of the two NHases was different, the NHase encoded by *B-A-P14K* was a little superior to that encoded by *A-B-P14K*.

For the two gene types of Co-NHases, cobalt incorporation is dependent on self-subunit swapping. The formation of a complex between the α-subunit and the self-subunit swapping chaperone is necessary for NHase maturation. The formation of a complex from an NHase with a gene organization of <β-subunit> <α-subunit> <P14K> appears more readily than that with a gene organization of <α-subunit> <β-subunit> <P14K> because the P14K gene is located just downstream of the α-subunit gene, and therefore the two subunits could easily interact with each other to form a complex after their translation. As a result, the catalytic efficiency of the NHase encoded by *B-A-P14K* was a little superior to that encoded by *A-B-P14K* (Fig. 8B).

The cloning and expression of a protein are direct methods for protein analysis. P14K was hardly detected on SDS-PAGE in a previous report [32]; similar phenomena have been found with other activation proteins (most likely self-subunit swapping chaperones) corresponding to other NHases. The addition of a Strep-tag N-terminal to P14K resulted in the successful expression of P14K. This improvement may be due to the N-end rule for the *E. coli* expression system [35]. Clarifying the mechanism of P14K overexpression is a topic for further study, which would be useful for the analysis of other proteins that are difficult to express.
